# Identification of a pathogenic *NHLRC1* variant in a consanguineous Pakistani family affected with severe and rapidly progressive Lafora disease

**DOI:** 10.1186/s42494-024-00193-0

**Published:** 2025-01-17

**Authors:** Zain Aslam, Bibi Zubaida, Ranjha Khan, Mazhar Badshah, Muhammad Naeem

**Affiliations:** 1 Medical Genetics Research Laboratory, Department of Biotechnology, Quaid-i-Azam University, Islamabad, 45320 Pakistan; 2 Department of Neurology and Neurophysiology, Pakistan Institute of Medical Sciences, Islamabad, 04485 Pakistan

**Keywords:** Progressive myoclonic epilepsy, Lafora bodies, Laforin, DNA sequencing, Mutation

## Abstract

**Background:**

Autosomal recessively inherited progressive myoclonic epilepsy of Lafora, which is also known as Lafora disease, is a fatal neurodegenerative disorder. It affects individuals in late childhood or early adolescence and presents with symptoms of progressive mental and physical deterioration. This disease is caused by pathogenic genetic variants in either of two genes: *EPM2A* on chromosome 6q24, encoding laforin, or *NHLRC1 (EPM2B)* on chromosome 6p22, encoding malin.

**Case presentation:**

In this study, we report a clinical and molecular investigation of Lafora disease segregating in a consanguineous Pakistani family. The proband presented with symptoms of rapidly progressive myoclonic epilepsy, but laboratory studies were negative for Lafora bodies and ragged red fibres. Sanger DNA sequencing of the genomic DNA of the proband for *EPM2A* and *NHLRC1* identified a previously reported pathogenic nonsense variant in *NHLRC1* (NM_198586.3:c.793C > T), which encodes the E3 ubiquitin ligase called malin. The *NHLRC1* variant was found in a homozygous state in the proband, predicting a premature stop codon at position 265 (NP_940988.2:p.Arg265Ter). If the mRNA escaped nonsense-mediated decay, it would result in a truncated protein lacking 130 amino acids, including three NHL (NCL-1, HT2A, LIN-41) repeats.

**Conclusions:**

Our study emphasizes the role of molecular genetic testing in patients presenting with symptoms of progressive myoclonic epilepsy.

## Background

Progressive myoclonic epilepsy (PME) is a group of uncommon clinically and genetically heterogeneous disorders including Lafora disease (LD), myoclonic epilepsy with ragged red fibers (MERRF), dentatorubral-pallidoluysian atrophy, sialidosis type 1 and type 2, several forms of neuronal ceroid lipofuscinosis and Unverricht-Lundborg disease. PME is characterized by the presence of the triad, including myoclonus, generalized epilepsy and progressive neurological deterioration [1].

LD, first described in 1911 by Gonzalo Lafora, is an ultra rare and fatal form of PME [2]. LD is characterized by the onset of symptoms in late childhood or adolescence, myoclonus, generalized tonic-clonic or occipital seizures, and progressive neurologic deterioration including ataxia and dementia [3]. The median time of survival of LD patients is approximately 11 years, while the median time of loss of autonomy is about six years from the disease onset [2].

The estimated frequency of LD is less than one case per million individuals worldwide. It follows an autosomal recessive mode of inheritance and is more prevalent in countries with high rates of consanguineous marriages [4]. However, the exact prevalence of LD in Pakistani population is unknown due to a lack of disease databases and the unavailability of molecular genetic testing except for a few specific diseases.

Currently, there is no cure for LD. Antiseizure medications (ASMs) can partially control the severity and frequency of seizures, but no effective therapy specifically for LD has been approved yet. Promising new therapeutic strategies for LD are being tested in animal models and human safety trials for gene therapy targeting in LD are currently in the planning stage [5].

LD is caused by pathogenic genetic variants transmitted autosomal recessively from parents to offspring in either of two genes: *EPM2A* on chromosome 6q24 encoding laforin (myoclonic epilepsy of Lafora-1; MIM 254780) or *NHLRC1/EPM2B* on chromosome 6p22 encoding malin (myoclonic epilepsy of Lafora-2; MIM 620681) [6, 7]. Among 298 patients with LD described in the literature, 44% had mutations in *EPM2A* and 56% had mutations in *EPM2B* [2]. The *EPM2A* gene consists of five exons, while *NHLRC1* is an intronless gene.

Malin (E3 ubiquitin protein ligase 1) is a 395-amino acid protein containing a RING domain in its N-terminal region and six NHL (NCL-1, HT2A, LIN-41) repeats domains in its C-terminal region. Laforin (laforin glucan phosphatase) is a 331-amino acid protein containing an N-terminal carbohydrate-binding domain and a C-terminal dual-specificity phosphatase domain. Both laforin and malin are involved in glycogen metabolism, and their interaction is supported by many studies. Although the exact mechanisms are not yet fully understood, it is likely that a functional laforin-malin complex is involved in processes that prevent the accumulation of insoluble glycogen-like particles. Pathogenic variants in *EPM2A*/*NHLRC1* result in the loss of function of laforin or malin, leading to poorly branched, hyperphosphorylated glycogen that precipitates in the form of Periodic acid-Schiff (PAS)-positive Lafora bodies. These intracellular inclusions accumulated in most tissues (such as brain, muscle, liver and skin) of affected individuals, contributing to neurodegeneration and neurological disease [8, 9].

More than 200 pathogenic variants in the *EPM2A*/*NHLRC1* have been reported so far, according to The Human Gene Mutation Database (available at https://www.hgmd.cf.ac.uk/ac/index.php; accessed on 11th October 2024). These variants include various types such as missense, nonsense, larger deletions, splicing and frameshift variants, and they are distributed across both genes.

In the present study, we investigated a four-generation Pakistani family segregating autosomal recessive LD in two siblings and identified a pathogenic nonsense variant in *NHLRC1*.

## Case presentation

### Methods

**Fig. 1:**
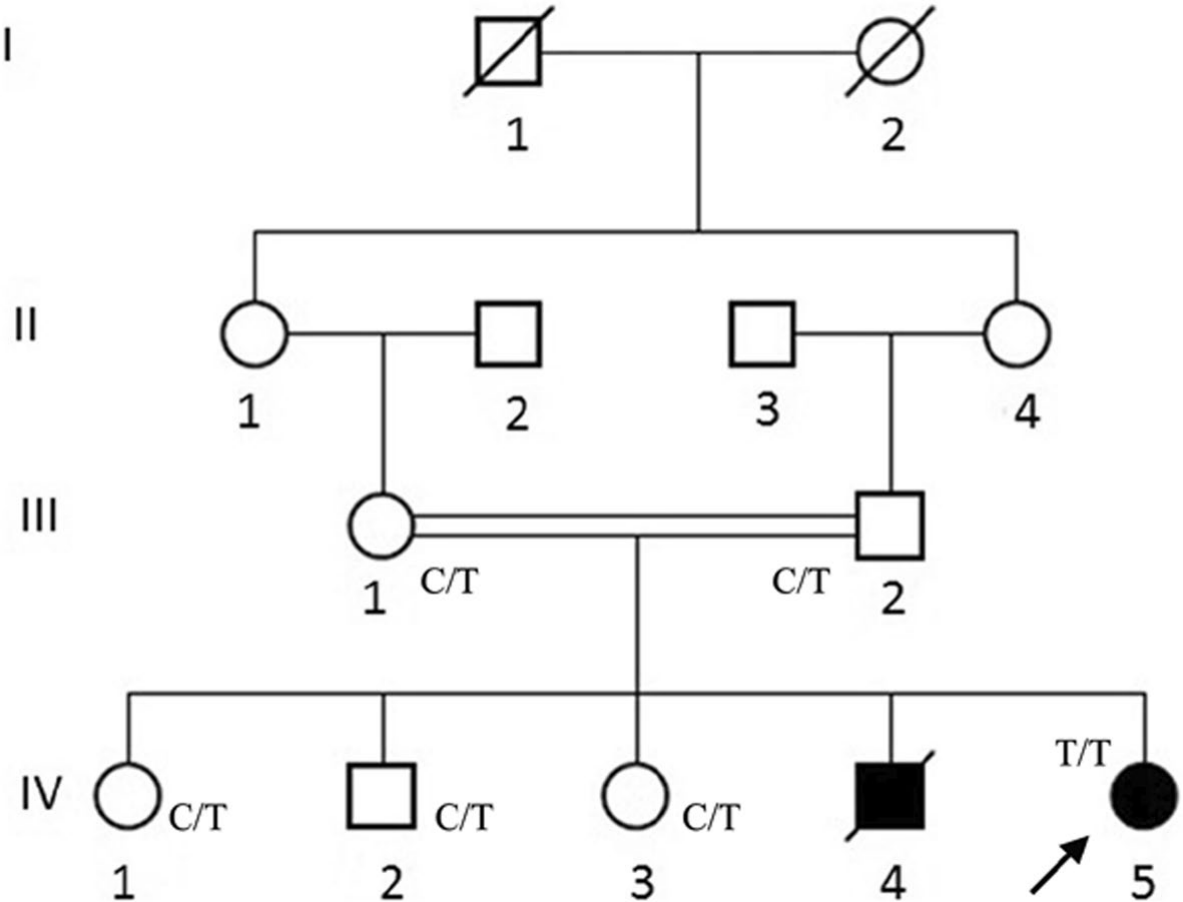
The pedigree of the Pakistani family affected with Lafora disease. A four-generation pedigree with two affected children born to consanguineous parents suggest autosomal recessive segregation of the epileptic phenotype. According to the DNA sequencing results (shown in Fig. 2), the inheritance of the *NHLRC1* mutant allele c.793C > T is mentioned alongside symbols of the individuals available for molecular study: the proband (IV-5), her normal parents (III-1 and III-2) and three normal siblings (IV-1, IV-2, and IV-3). The elder affected sibling (IV-4) of the proband was not available for the study due to his death at the age of 16. In the pedigree, males are represented by squares, females by circles, affected persons by the filled symbols, and deceased ones by a diagonal line through the symbols. The proband is indicated by an arrow

Approval for the study was obtained from the Institutional Review Board of Quaid-i-Azam University, Islamabad (QAU/DFBS/216). An informed consent form was signed by all individuals participating in the study or their guardians (in the case of minors). Clinical studies and diagnostic tests were carried out at the Pakistan Institute of Medical Sciences Islamabad. Peripheral blood (5 ml) was drawn in EDTA collection tubes from six subjects, including one affected female (IV-5), her parents (III-1, III-2) and three unaffected siblings (IV-1, IV-2, IV-3), and stored at 4 °C (Fig. 1). One affected individual (IV-4) had passed away by the time of study, and therefore a blood/DNA sample was not available for molecular investigation. However, clinical history was recorded from limited medical records available from the family.

Genomic DNA extraction from the blood of affected and normal individuals was performed with QIAamp DNA Blood Maxi Kit (Qiagen, Hilden, Germany). The quantity and quality of the extracted DNA were tested by spectrophotometric measurement of absorbance at 260 nm and 280 nm using a Quantus Fluorometer (Promega Corporation, Madison, WI), and the DNA was diluted to 50 ng/µL for amplification by polymerase chain reaction (PCR). To screen for mutations, human mitochondrial DNA amplification was performed with the help of 22 primer sets designed previously [10] while primers for the amplification of exons and splice junctions of *EPM2A* and *NHLRC1* genes were manually designed and are available upon request. PCR amplification was conducted according to a standard procedure in a total volume of 50 µL, which included 2X GoTaq^®^ Green Master Mix (Promega Corporation, Madison, WI), 50 ng of genomic DNA and 20 pmoles of each primer. The PCR was carried out for 35 cycles, with the following thermal cycling conditions: 95 °C for 30 s, an annealing temperature for 30 s, 72 °C for one minute, followed by final extension at 72 °C for seven minutes in MultiGene OptiMax Thermal Cycler (Labnet International USA). The PCR products were purified using the GeneJET PCR Purification Kit No. K0702 (Thermo Fisher Scientific Inc. USA) and subsequently subjected to sequencing in Applied Biosystems automatic sequencer ABI3730xL, using Big Dye terminator cycle sequencing kit V3.1 (PE Applied Biosystems, Foster City, CA). BioEdit Sequence Alignment Editor version 7.2.6 was used to analyse the sequenced amplicons.

Amino acid conservation analysis of the protein variant was performed with the help of COBALT (Constraint-based Multiple Alignment Tool; available at https://www.ncbi.nlm.nih.gov/tools/cobalt/re_cobalt.cgi). In-silico 3D structures were predicted with the help of I-Tasser software (https://zhanggroup.org/I-TASSER/). The pathogenicity of the identified variant was interpreted following the guidelines and standards set by the American College of Medical Genetics and Genomics (ACMG) and Association for Molecular Pathology (AMP).

## Results

The proband (IV-5) presented with symptoms of progressive myoclonic epilepsy, starting as febrile fits at the age of 14. She developed myoclonia in her upper and lower extremities and started having generalized tonic-clonic seizures by the age of 15. Her symptoms manifested with general clumsiness, inability to move, headaches, dizziness, generalized body stiffness, aphasia and ataxia. Over the course of two years, her symptoms progressively worsened, characterized by increased seizure intensity and duration, along with gradually diminished mental capacity and motor skills. At the age of 16, her demeanor was withdrawn, and she remained in a vegetative, unresponsive state while bedridden, requiring assistance for several bodily functions. Her electroencephalogram (EEG) revealed generalized spikes and background slowing; microscopic examination of skin biopsy with PAS staining was negative for Lafora bodies. Enzyme histochemistry was also negative for ragged red fibres. She had one affected brother (IV-4) who had similar clinical presentation and progression but with an earlier disease onset (at eight years of age). He died at 16 after being hospitalized for complications related to his digestive system, stroke and respiratory failure. However, the role of LD in the underlying cause of his death could not be established due to the unavailability of complete medical history of the patient (IV-4).

The clinical presentation of the proband (IV-5) was suggestive of PME. Considering her symptoms, such as myoclonus, ataxia, seizures, and the absence of Lafora bodies in the skin biopsy, she was initially diagnosed with MERRF by local clinicians. However, owing to consanguinity, a recessive pattern of inheritance and clinical features overlapping with LD, we considered to conduct parallel screening of the proband for both the mitochondrial genome and LD genes (*EPM2A* and *NHLRC1*).

The results of the mitochondrial DNA sequencing revealed no pathogenic variants in *MT-TK* or *MT-TL *genes. Owing to the genetic heterogeneity of the disorder, we examined the variants that were involved in cerebral pathologies and encephalopathies and checked their segregation in the mother (III-1) of the proband (IV-5). However, the identified candidate variants were excluded as the underlying cause of the phenotype in the patient.

**Fig. 2:**
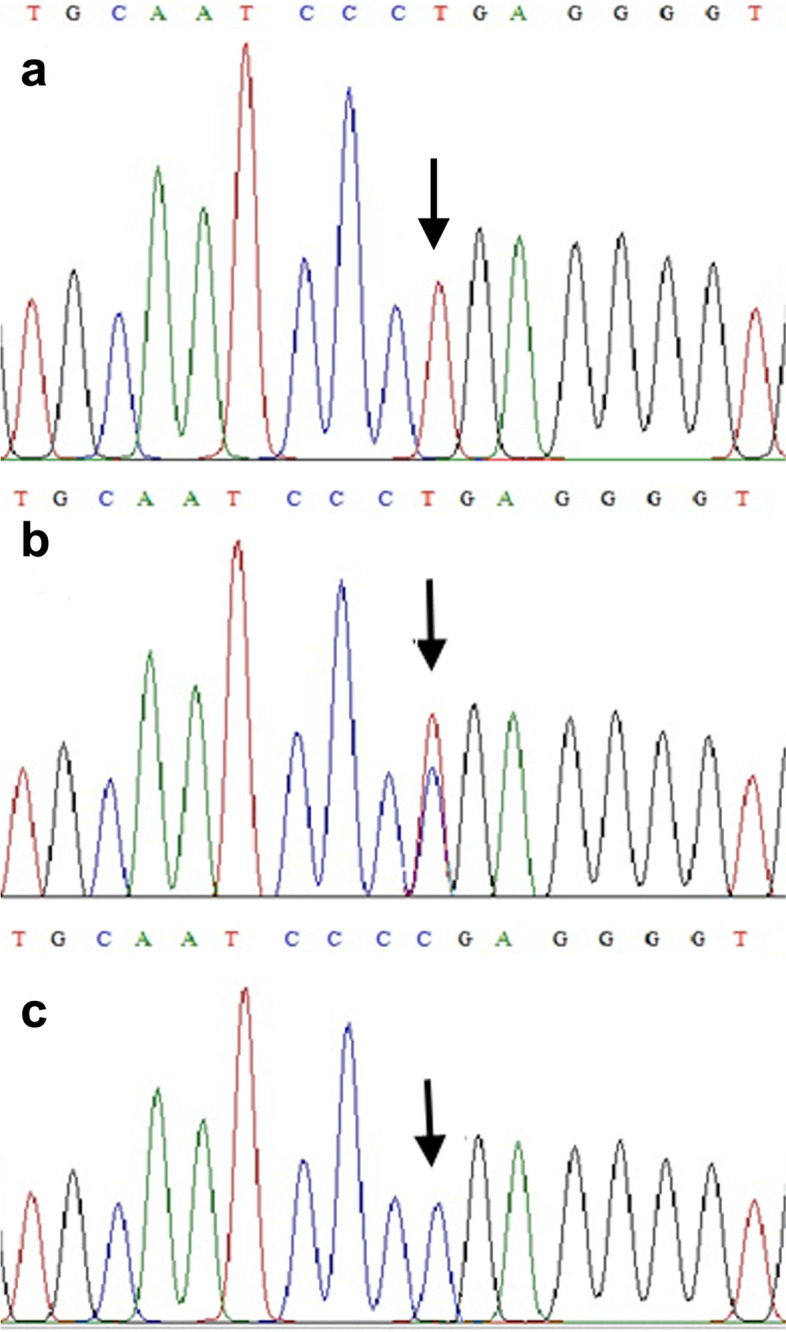
The electropherograms show the results of *NHLRC1* genomic DNA sequencing in the Pakistani family affected with Lafora disease. **a** The homozygous c.793C > T variant is identified in the proband (IV-5). **b** Heterozygous c.793C > T variants were found in the normal parents (III-1 and III-2) and three normal siblings (IV-1, IV-2, and IV-3). **c** A homozygous wild-type sequence is shown from a normal individual from outside the affected family

DNA sequencing of *EPM2A* and *NHLRC1* in the proband identified a pathogenic nonsense variant in *NHLRC1* (NM_198586.3:c.793C > T) in the homozygous state (Fig. 2a), predicting premature truncation of the protein due to a stop codon at amino acid position 265 (NP_940988.2:p.Arg265Ter). If the mRNA were to escaped nonsense-mediated decay, it would result in a protein lacking 130 amino acids, including three NHL repeats. This variant has been previously reported in a Turkish patient presenting with initial clinical symptoms similar to those of this proband (IV-5) [11].

The single coding exon of *NHLRC1* was PCR amplified with the gDNA from the normal parents (III-1 and III-2) and the normal siblings (IV-1, IV-2, and IV-3) of the proband (IV-5) and subjected to Sanger sequencing. The variant c.793C > T was found in the heterozygous state in these individuals, confirming its segregation with the disease phenotype in the family (Fig. 2b).

**Fig. 3:**
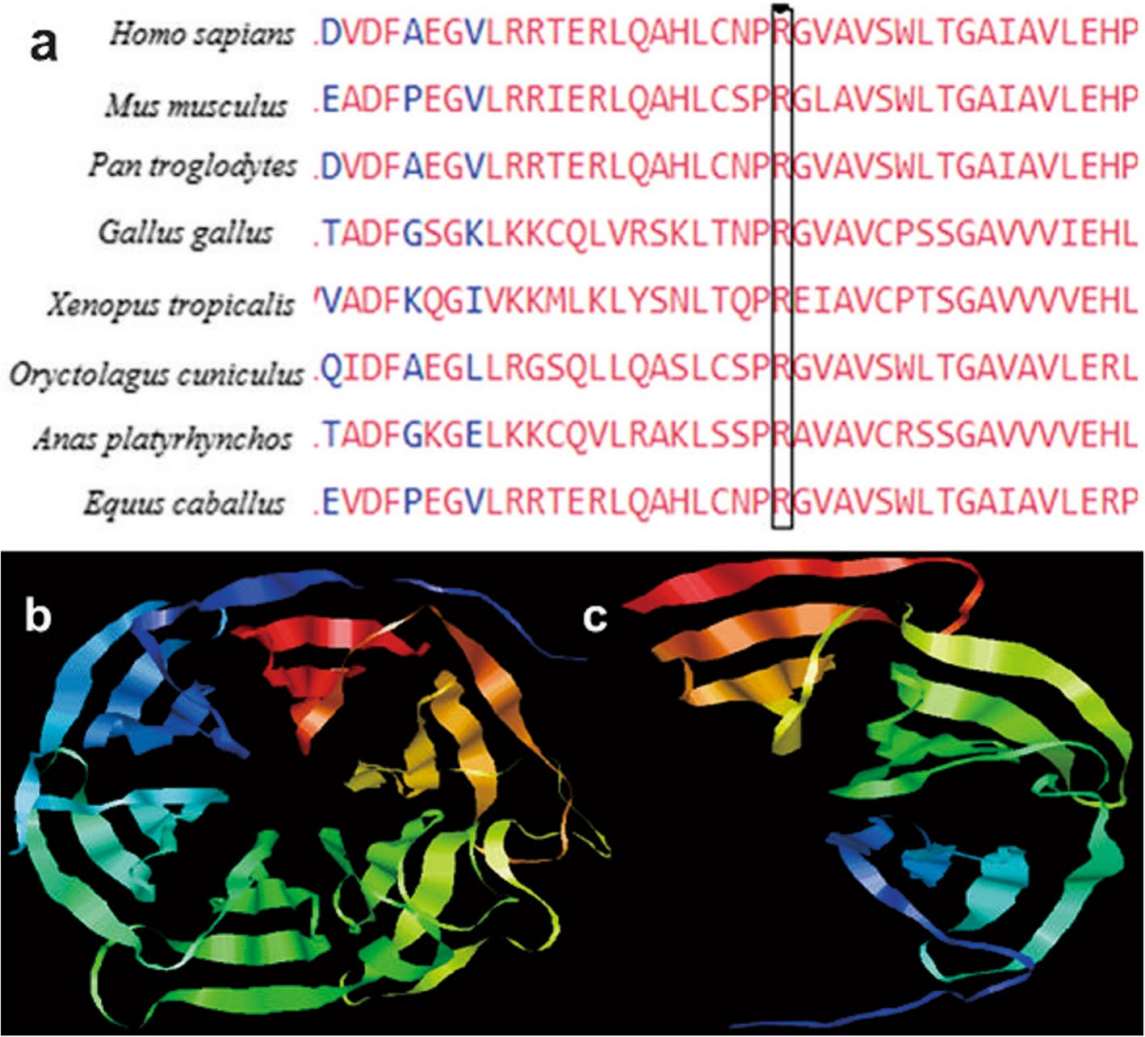
In-silico analysis of the NHLRC1 protein. **a** Multiple sequence alignment of the NHLRC1 with COBALT shows amino acid p.Arg265 is highly conserved among vertebrate species; **b** I-Tasser predicted 3D structure of the normal NHLRC1 protein; **c** The mutated p.Arg265Ter NHLRC1 protein shows a loss of three NHL-repeats

Amino acid conservation analysis performed with COBALT showed that the p.Arg265 amino acid of NHLRC1 protein was highly conserved among vertebrate species (Fig. 3a). The three-dimensional structure predictions of the normal and mutated c.793C > T NHLRC1 through I-Tasser showed that the stop-gained p.Arg265Ter variant resulted in the loss of three NHL-repeats in the translated protein (Fig. 3b and c). According to the ACMG and AMP guidelines, the c.793C > T variant was clinically interpreted as "pathogenic" (PVS1 PM2 PP1 PP5).

## Discussion

In this study, we investigated a four-generation Pakistani family segregating autosomal recessive LD in two siblings. We enrolled six family members in the study: one affected individual (IV-5), and five unaffected family members (III-, III-2, IV-1, IV-2, IV-3). Molecular genetic analysis through direct DNA sequencing identified a pathogenic nonsense variant p.Arg265Ter in *NHLRC1* segregating in an autosomal recessive mode of inheritance with the disease phenotype in the family. In-silico analysis and clinical interpretation following ACMG guidelines support the pathogenicity of the identified variant.

LD phenotypes caused by genetic defects in *EPM2A* and *NHLRC1* are clinically indistinguishable, and genotype-phenotype correlations are difficult to establish. *NHLRC1* variants are usually associated with a slower progression of the disease or adult onset with a delayed age of death compared with patients with *EPM2A* mutations [11]. However, a few patients with *NHLRC1* variants and rapidly progressive disease course have been reported (including the patients with the p.Arg265Ter variant presented in the current study). Therefore, *NHLRC1* variants are not necessarily associated with slow progression [12]. Recently published prognostic meta-analyses of patient-level data also indicated that the type of mutated gene (*EPM2A* or *NHLRC1*) is not a prognostic factor in LD. But biallelic truncating variants in the *NHLRC1* are significantly associated with a worse prognosis in terms of survival and loss of autonomy. The truncating variants resulting in lower molecular weight or absent proteins may have more deleterious effects, leading to a more severe phenotype [2, 9].

The current study illustrates the variable expression of the p.Arg265Ter variant. The patient IV-4 of our family presented an earlier age of disease onset (eight years old) than the proband, IV-5 (14 years old). However, both had rapid disease progression: IV-4 died within eight years of disease onset, whereas IV-5 was completely bedridden within two years of disease onset. LD patients with the identical p.Arg265Ter variant in a previous report presented with onset at 13 years of age but exhibited a milder and longer course of the disease [11]. The intrafamilial and interfamilial phenotypic variability among patients harbouring identical pathogenic variant may be explained through modifier genes.

LD has a severe prognosis, rendering the patients bedridden, and leading to death within a few years from disease onset. Early diagnosis and intervention are crucial for improving outcomes in LD patients. The presence of parental consanguinity and Lafora bodies are significant in confirming the diagnosis of LD. Lafora bodies usually accumulate in the skin, muscle, liver, and brain tissues of patients with LD. Therefore, the diagnosis can be confirmed by performing a biopsy from any of these organs, although the most used and accessible site with high yield is the axillary skin region. In our patient (IV-5), Lafora bodies were not observed during histopathological examination of a PAS-stained skin biopsy, although parental consanguinity was established. In a previous study, two siblings with progressive myoclonus epilepsy presented with typical symptoms of LD, but their parents were not consanguineous, and skin biopsies were negative for Lafora bodies. However, a recurrent homozygous *NHLRC1* frameshift variant was identified in the gDNA of these patients through whole-exome sequencing [13].

Recent studies have indicated Lafora bodies were detected in 120 out of 138 (87%) skin biopsies from genetically confirmed LD patients [2]. However, patients with early childhood onset LD have also presented with negative skin biopsies (but positive muscle) for Lafora bodies. Furthermore, sweat glands in skin biopsies have PAS-D positive normal secretory polysaccharide contents and are falsely labelled as Lafora bodies in many cases [8]. Considering the potential for false negative and false positive results in skin biopsies, molecular testing is recommended as the first-line diagnostic test in all cases where PME is suspected [1].

## Conclusions

Our study illustrates the clinical heterogeneity and variable expression of the *NHLRC1* variant p.Arg265Ter underlying myoclonic epilepsy of Lafora-2. Our findings emphasize the role of implementing high-yield molecular genetic testing (PME gene panels or preferably whole exome sequencing combined with in-silico analysis and clinical data) for early, non-invasive and accurate diagnosis of epileptic disorders to end the diagnostic odyssey endured by many patients and their families. Improved diagnostic strategies in LD are also crucial to design studies on the effectiveness of upcoming new disease-modifying therapies that will soon be employed in clinical trials.

## Data Availability

Not applicable.

## Abbreviations

ACMG: American College of Medical Genetics and Genomics
AMP: Association for Molecular Pathology
COBALT: Constraint-based Multiple Alignment Tool
LD: Lafora disease
MERRF: Myoclonic epilepsy with ragged red fibers
PAS: Periodic acid-Schiff
PCR: Polymerase chain reaction
PME: Progressive myoclonic epilepsy
